# Cement/Sulfur for Lithium–Sulfur Cells

**DOI:** 10.3390/nano14040384

**Published:** 2024-02-19

**Authors:** Tzu-Ming Hung, Cheng-Che Wu, Chung-Chan Hung, Sheng-Heng Chung

**Affiliations:** 1 Department of Materials Science and Engineering, National Cheng Kung University, No. 1, University Road, Tainan City 70101, Taiwan; 2 Department of Civil Engineering, National Cheng Kung University, No. 1, University Road, Tainan City 70101, Taiwan; 3 Hierarchical Green-Energy Materials Research Center, National Cheng Kung University, No. 1, University Road, Tainan City 70101, Taiwan

**Keywords:** cement, sulfur, lithium–sulfur battery, electrochemistry, sustainable energy

## Abstract

Lithium–sulfur batteries represent a promising class of next-generation rechargeable energy storage technologies, primarily because of their high-capacity sulfur cathode, reversible battery chemistry, low toxicity, and cost-effectiveness. However, they lack a tailored cell material and configuration for enhancing their high electrochemical utilization and stability. This study introduces a cross-disciplinary concept involving cost-efficient cement and sulfur to prepare a cement/sulfur energy storage material. Although cement has low conductivity and porosity, our findings demonstrate that its robust polysulfide adsorption capability is beneficial in the design of a cathode composite. The cathode composite attains enhanced cell fabrication parameters, featuring a high sulfur content and loading of 80 wt% and 6.4 mg cm^−2^, respectively. The resulting cell with the cement/sulfur cathode composite exhibits high active-material retention and utilization, resulting in a high charge storage capacity of 1189 mA∙h g^−1^, high rate performance across C/20 to C/3 rates, and an extended lifespan of 200 cycles. These attributes contribute to excellent cell performance values, demonstrating areal capacities ranging from 4.59 to 7.61 mA∙h cm^−2^, an energy density spanning 9.63 to 15.98 mW∙h cm^−2^, and gravimetric capacities between 573 and 951 mA∙h g^−1^ per electrode. Therefore, this study pioneers a new approach in lithium–sulfur battery research, opting for a nonporous material with robust polysulfide adsorption capabilities, namely cement. It effectively showcases the potential of the resulting cement/sulfur cathode composite to enhance fabrication feasibility, cell fabrication parameters, and cell performance values.

## 1. Introduction

The growing global demand for electric vehicles and large-scale energy storage has highlighted the significant challenge of sustainably sourcing the battery technology necessary for transitioning to renewable electricity. This technology must possess high energy density and be cost-effective and environmentally friendly. Lithium–sulfur cells offer a solution for developing batteries that not only outperform current options but also utilize readily available materials. They have the potential to increase energy density by 3–5 times compared with current lithium-ion batteries and can be prepared using inexpensive and non-toxic raw materials [1,2,3,4]. However, introducing sulfur into a lithium cell poses a challenge because of an inefficient and irreversible electrochemical reaction between solid-state and liquid-state active materials during cell discharge and charge processes [3,4]. The sulfur cathode forms sulfur and lithium sulfide as solid-state active materials in the full charge and discharge stages, respectively. These electrochemical end-products exhibit high resistance, resulting in poor reaction kinetics and high polarization. Thus, the sulfur cathode experiences limited utilization and an insufficient amount of active material, affecting both cell performance and design [5,6,7,8]. In intermediate discharge/charge stages, polysulfides are generated with different and complex formulas, such as Li_2_S_x_ with x = 4 − 8. These polysulfides have high solubility in the liquid electrolyte, leading to the uncontrollable loss of the active material and the degradation of both the electrolyte and electrodes [9,10,11,12]. Because of this adverse reaction, the components and configuration of lithium–sulfur cells have been designed to accommodate the unique solid–liquid–solid redox conversion electrochemistry [5,6,7,8,9,10,11,12,13].

The primary challenge in achieving high-performance lithium–sulfur cells is the complex and multistep redox reaction that occurs within the high-capacity sulfur cathode. To stabilize the sulfur cathode and enhance its electrochemical efficiency, studies have mainly focused on modification of the cathode. This modification aims to harness the high charge storage capacity and efficient conversion chemistry of the battery to address disadvantages such as high resistance, rapid capacity degradation, and irreversible solid–liquid–solid conversion [10,11,12,13]. The focus of cathode modification in lithium–sulfur battery research is to create a porous matrix with high conductivity to accommodate the active material [14,15,16] and improve the redox capability of the trapped active material during charge and discharge processes [17,18,19]. In terms of cathode materials, various porous carbon materials have been identified for their superior electron transfer capabilities and effective active-material retention properties [15,20,21,22]. Carbon materials, exhibiting high conductivity in the range of 10^2^–10^6^ S cm^–1^, effectively counteract the high resistance of the active material, thereby enhancing the electrochemical utilization of sulfur. Microporous and mesoporous carbons contribute to polysulfide retention and lithium-ion transfer, respectively. Additionally, a large pore volume and a well-defined porous network further enhance the high loading capacity of the sulfur cathode [19,23,24,25]. Other material designs include functional polymer frameworks and oxide additives for effective polysulfide trapping, catalyst additives for the rapid activation and conversion of active materials, and sulfur-based compounds that exhibit strong reaction activity as polysulfide catholytes or limited polysulfide diffusion as sulfurized polyacrylonitrile and organosulfur [17,18,26,27,28]. In terms of cathode structures, sulfur-based cathode composites have gained prominence. These cathodes employ the aforementioned materials as host materials to encapsulate and trap the active material, resulting in a high reversible capacity and utilization of sulfur [15,26,27,28,29]. In addition to the synthesis of sulfur-based composites, porous current collectors have been developed to replace flat and heavy aluminum foil current collectors. These current collectors have a porous network, layered structure, and core–shell configuration, enabling the encapsulation of the active material in both solid and liquid states through various methods, such as melting, pressing, injecting, and casting [29,30,31]. The modification of the cathode, involving adjustable materials and structures along with their corresponding preparation methods, has become the focus in the development of lithium–sulfur battery cathodes.

Inspired by the abovementioned progresses, this study explored the potential of cement, globally recognized as the second most utilized material after water, for synthesizing a novel cement/sulfur energy storage composite. This composite uniquely combines cement’s structural properties with sulfur to create a hosting matrix for the active material. While substituting carbon with cement may not lead to a significant reduction in cell resistance, the use of nonporous cement brings notable benefits. It possesses robust polysulfide adsorption capability and low electrolyte consumption, thereby improving performance throughout cell cycling. This approach leverages the ubiquity and physical characteristics of cement to innovate in the field of energy storage. The robust stabilization of the active material allowed us to achieve a high sulfur content of 80 wt% in the cement/sulfur energy storage material. This high-sulfur-content energy storage material was then used to design a high-loading sulfur cathode, achieving a sulfur loading of up to 6.4 mg cm^−2^. The large amount of sulfur in the cement/sulfur cathode that had high electrochemical utilization resulted in a high charge storage capacity of 1189 mA∙h g^−1^ and a high rate performance from C/10 to C/3 rates with a long cycle life of 200 cycles. Material and electrochemical analyses indicated that the cement in the cement/sulfur energy storage material was highly effective in trapping polysulfides. Thus, the cement/sulfur energy storage material retained polysulfides effectively, stabilizing them within the cathode. This stabilization facilitates the activation and utilization of sulfur, ensuring that the large amount of sulfur is effectively used electrochemically. This results in a high cathode performance, achieving a high peak areal capacity of 7.61 mA∙h cm^−2^, a high gravimetric capacity of 951 mA∙h g^−1^, and an energy density of 15.98 mW∙h cm^−2^. The electrochemical performance meets the requirements for various applications, such as in electric vehicles, exhibiting an energy density superior to that of conventional oxide cathodes [31,32,33,34].

## 2. Materials and Methods

### 2.1. Preparation of Cement/Sulfur Cathode Composite

The cement/sulfur energy storage material was synthesized using a facile melting method with a mixture of 80 wt% sulfur and 20 wt% cement (Portland cement, Universal Cement Corporation, Taipei, Taiwan) at 155 °C for 6 h to yield the cement/sulfur energy storage material with sulfur covering the cement particles. The prepared cement/sulfur energy storage material was dispersed in a blank electrolyte containing 1.85 M LiC_2_F_6_NO_4_S_2_ (98%, Acros Organics, Thermo Fisher Scientific, Waltham, MA, USA) and 0.1 M LiNO_3_ (99+%, Acros Organics) in a mixed solvent of C_4_H_10_O_2_ (99+%, Acros Organics) and C_3_H_6_O_2_ (99.8%, Acros Organics). This dispersion was drop-casted onto a carbon-paper current collector to form a cement/sulfur cathode composite with a sulfur content and loading of 80 wt% and 6.4 mg cm^−2^, respectively. A reference cathode was prepared by the same experimental processes, using 80 wt% sulfur and 20 wt% conductive carbon.

### 2.2. Materials and Chemical Characterization

A comprehensive analysis of the fundamental physicochemical characteristics of cement was conducted with X-ray diffraction (XRD), Raman spectroscopy, and nitrogen adsorption–desorption isotherms. XRD was performed using an X-ray diffractometer (D8 Discover with GADDS, Bruker, Billerica, MA, USA) with Cu Kα radiation in the range of 20° to 80°. Raman spectroscopy was performed using a Micro-Raman and Micro-PL spectrometer (Horiba Jobin Yvon LabRAM HR, Longjumeau, France) equipped with a 532 nm laser in the range of 300 to 1900 cm^−1^. Nitrogen adsorption–desorption isotherms were plotted using a gas sorption analyzer (Autosorb iQ, Anton Paar, Graz, Austria) at 77 K in relative pressures ranging from 10^−5^ to 10^0^ P/P_0_. These isotherms were used to calculate the specific surface area through the Brunauer–Emmett–Teller theory. Porosity and pore size were determined using Horvath–Kawazoe, density functional theory, and Barrett–Joyner–Halenda analyses. 

Polysulfide adsorption measurements were performed using dilute 0.001 M Li_2_S_6_ that was prepared by mixing elemental sulfur and lithium sulfide in a molar ratio of 5:1 in the blank electrolyte. The as-prepared dilute polysulfide was added with 80 mg of cement and rested for 7 days. A reference sample was maintained as a blank. After polysulfide adsorption measurements, the solutions were retrieved for ultraviolet–visible spectroscopy (UV–Vis, U4100, Hitachi, Tokyo, Japan) performed in the range of 250 to 600 nm. 

Thermogravimetric analysis (TGA, TGA4000, Perkin Elmer, Waltham, MA, USA) was conducted using the cement/sulfur energy storage material to determine the sulfur content in the composite from room temperature to 450 °C at a ramping rate of 5 °C min^−1^. Cement and the cement/sulfur energy storage material before and after cycling were examined using a high-resolution scanning electron microscope (SEM, SU8000, Hitachi). Elemental distribution within the materials was analyzed using an energy-dispersive X-ray spectrometer (EDS, XFlash 5010, Bruker).

### 2.3. Electrochemical and Cell Performance Characterization 

The electrochemical and cell performances of the cement/sulfur energy storage material were examined in a lithium–sulfur cell with a high-sulfur-loading cement/sulfur cathode composite that had a sulfur content and loading of 80 wt% and 6.4 mg cm^−2^, respectively. The lithium–sulfur cell was subsequently assembled. Its assembly included a separator (2500, Celgard, Charlotte, NC, USA), lithium metal serving as the counter and reference electrode, and an additional blank electrolyte added to maintain a low electrolyte-to-sulfur ratio of 8 µL mg^−1^. 

The low-electrolyte lithium–sulfur cell with the high-loading cement/sulfur cathode composite was analyzed using electrochemical impedance spectroscopy (EIS) before and after 200 cycles at a frequency range from 1 MHz to 1 mHz, cyclic voltammetry (CV) measurements within a voltage window of 1.5–2.8 V at scanning rates of 0.010–0.030 mV s^−1^, and battery tests (i.e., cyclability, rate performance, and discharge/charge voltage profiles) within a voltage window of 1.5–2.8 V at cycling rates of C/10 to C/3 in the constant rate testing for 200 cycles and C/20 to C/3 in the rate capability testing.

## 3. Results and Discussion

### 3.1. Material Characteristics

**Figure 1:**
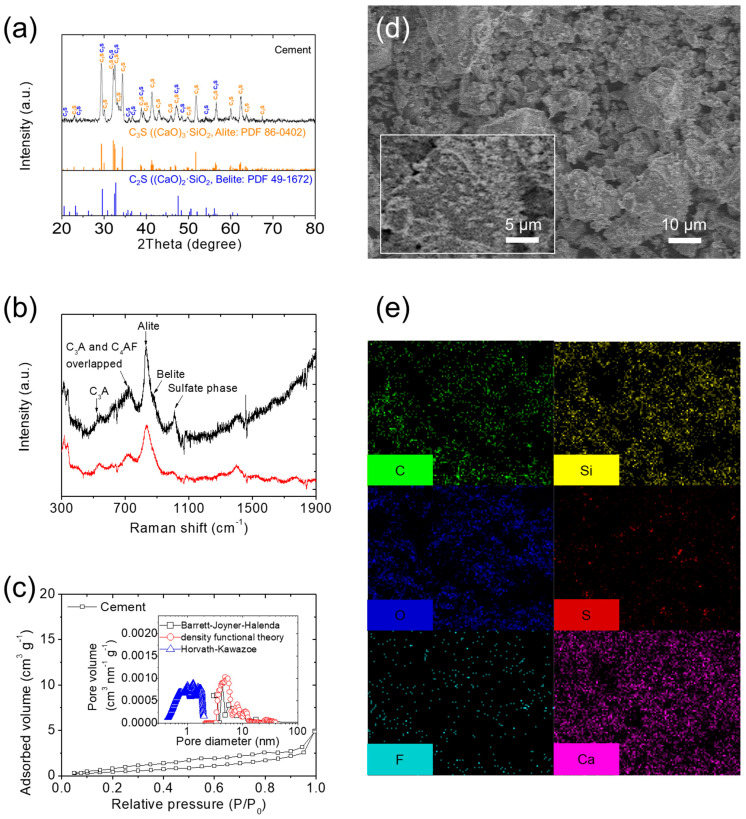
Material characteristics. (**a**) X-ray diffraction (XRD), (**b**) Raman spectroscopy (black: raw data; red: normalization by reducing the background), (**c**) nitrogen adsorption–desorption isotherms with pore size distribution, (**d**) scanning electron microscopy (SEM), and (**e**) energy-dispersive X-ray spectroscopy (EDS) of cement.

The fundamental material characteristics of the host material cement were examined. The findings are illustrated in Figure 1. XRD analysis indicated that the cement was made of hydraulic cement containing calcium silicates, specifically C_3_S (orange: (CaO)_3_·SiO_2_: Alite, PDF 86-0402) and C_2_S (blue: (CaO)_2_·SiO_2_: Belite, PDF 49-1672; Figure 1a). The XRD patterns revealed the presence of pure Portland cement, which will be utilized in subsequent lithium–sulfur cell studies. Raman spectroscopy analysis of the hydraulic cement showed major peaks at 834–848 and 846–864 cm^−1^, corresponding to C_3_S ((CaO)_3_·SiO_2_: Alite) and C_2_S ((CaO)_2_·SiO_2_: Belite), respectively, which are the main components of Portland cement. Additional peaks were identified at 504–510, 740–770, 750–760, and 1008–1020 cm^−1^, which corresponded to minor materials in cement, namely, C_3_A ((CaO)_3_·Al_2_O_3_), C_4_AF ((CaO)_4_·Al_2_O_3_·Fe_2_O_3_), C_3_A ((CaO)_3_·Al_2_O_3_), and gypsum (CaSO_4_·2 H_2_O), respectively (Figure 1b). The results of both XRD and Raman spectroscopy analyses confirmed that calcium silicates were the main materials in our cement substrate [35,36]. In addition to chemical analysis, physical analysis and nitrogen adsorption–desorption isotherms were conducted to examine the specific surface area and corresponding porosity analysis of the hydraulic cement. The hydraulic cement exhibited the adsorption and desorption behavior of a nonporous material with low gas adsorption. Thus, the corresponding pore size distribution exhibited limited nanoporosity, with a low specific surface area of 1.90 m^2^ g^−1^, an average pore diameter of 16.17 nm, and a low total pore volume of 0.01 cm^3^ g^−1^ (Figure 1c). Microstructural and elemental inspections, including SEM and EDS, were performed to observe the material’s physicochemical morphology and elemental distribution. SEM images revealed that the pristine cement was composed of micro-sized particles and clusters, with specific particle sizes of 10–30 µm (Figure 1d). The corresponding EDS analysis displayed the uniform distribution of the main constituent elements Si and Ca in the materials (Figure 1e). The comprehensive material physicochemical analysis confirmed the suitability of the nonporous, pristine hydraulic cement for modification of the cathode structure, leading to the formation of the first cement/sulfur cathode composite.

**Figure 2:**
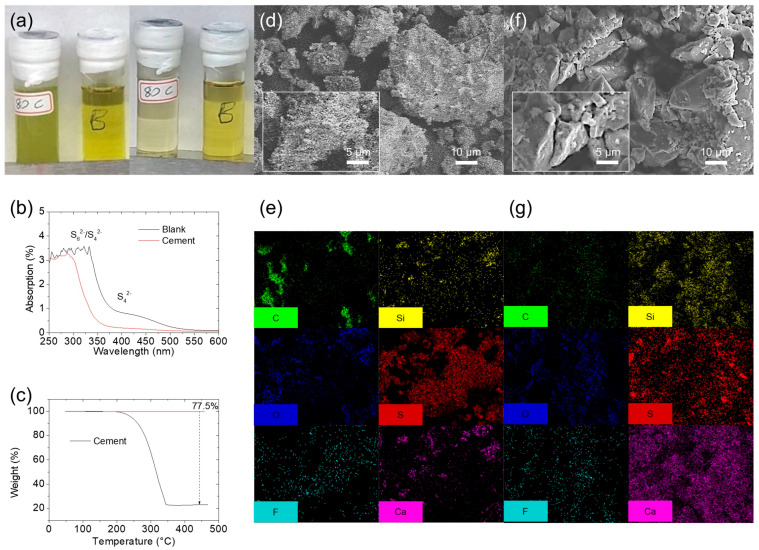
Material characteristics. (**a**) Polysulfide adsorption analysis and (**b**) ultraviolet–visible (UV–Vis) spectroscopy of cement. (**c**) Thermogravimetric analysis (TGA), (**d**) SEM, and (**e**) EDS of cement/sulfur energy storage material and (**f**) SEM and (**g**) EDS of the cycled cement/sulfur energy storage material.

Prior investigations into cathode materials have predominantly employed porous substrates to accommodate the active material and facilitate the physical absorption of diffusing polysulfides [19,20,21,22]. Apart from the physical adsorption enabled by porous substrates, certain materials exhibit the capability to chemically adsorb polysulfides at their surface, employing mechanisms such as Li-O and metal-S bonding in metallic oxides, showcasing robust polysulfide retention [17,18,19,35,37]. However, distinguishing between physical absorption and chemical adsorption has proven challenging in many reported studies due to their intertwined nature. To address this issue, the utilization of nonporous hydraulic cement, which precludes physical absorption, has provided a conducive platform for delving into the chemical adsorption of diffusing polysulfides, as summarized in Figure 2.

The chemical polysulfide adsorption capability of the nonporous hydraulic cement was initially studied. Polysulfide adsorption measurements revealed that a dilute polysulfide solution mixed with cement became transparent after resting for 7 days, whereas a reference dilute polysulfide solution retained its original color. These findings indicated the nonporous cement’s ability to trap polysulfides through chemical adsorption (Figure 2a). Subsequently, UV–Vis analysis of the retrieved polysulfide solution was conducted. UV–Vis spectroscopy revealed no detectable polysulfide absorption in the retrieved polysulfide solution containing cement. However, the blank polysulfide solution showed strong and distinct S_6_^2−^/S_4_^2−^ and S_4_^2−^ polysulfide peaks. This comparison provided solid evidence of the nonporous cement’s effective polysulfide retention (Figure 2b) [26,27,28,29]. 

Based on these positive attributes of cement, it was used as the host material in the fabrication of the cement/sulfur energy storage material through a facile melting method at 155 °C for 6 h. During sample preparation, the melted sulfur with low viscosity covered the cement particles, effectively trapping them within the host substrate. This arrangement allowed the trapped sulfur to remain in contact with the electrolyte and the current collector in the cathode. The resulting cement/sulfur energy storage material was analyzed through TGA. The results of TGA indicated a weight loss of 78 wt% at 340 °C, consistent with the sample composition of 80 wt% sulfur and 20 wt% cement (Figure 2c). This high sulfur content in the cement/sulfur energy storage material suggested its high practical capacity in lithium–sulfur cells. Next, the morphology and elemental composition of the cement/sulfur energy storage material before and after cycling in the lithium–sulfur cell were compared. The freshly prepared cement/sulfur cathode composite displayed a fluffy surface morphology and intense elemental sulfur signals, indicative of the surface coating of sulfur on cement particles (Figure 2d,e). After cycling, the cycled cement/sulfur cathode composite showed a morphology of particles that closely resembled the initial state. Moreover, the elemental distribution of the materials remained nearly unchanged, indicating the stability of the composite structure. The robustness of the cement/sulfur energy storage material in retaining polysulfides within the cathode region was confirmed by the pronounced elemental sulfur signal. Additionally, the absence of a thick and loose surface covering layer of re-deposited sulfide further demonstrated the effectiveness of the material in trapping polysulfides at the cement surface and therefore stabilizing them in the cathode region. Thus, the cycled cathode remained free from the ionic/electronic insulating sulfide layer generated by the precipitation of diffusing polysulfides (Figure 2f,g).

### 3.2. Electrochemical Analysis

**Figure 3:**
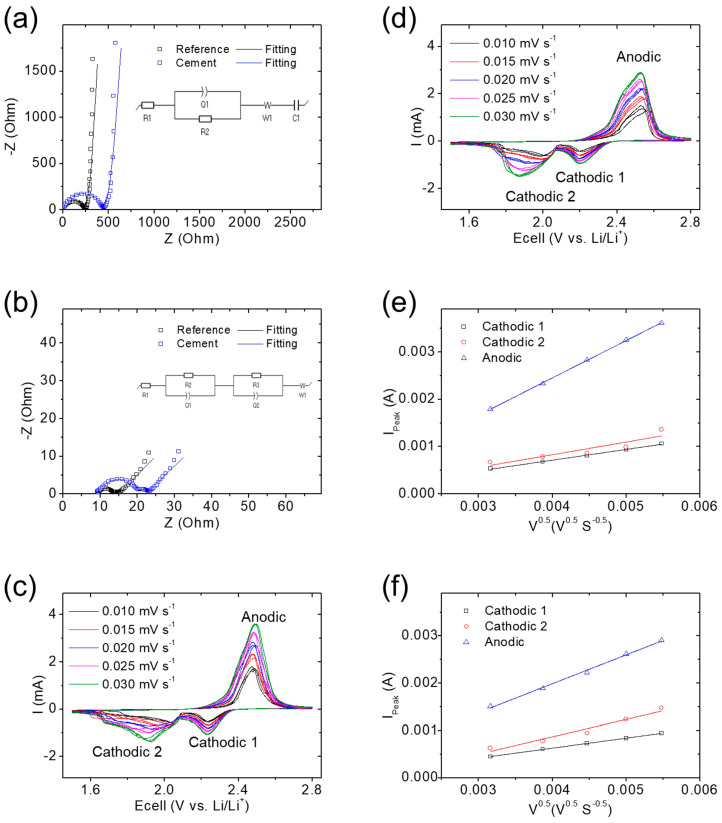
Electrochemical analysis. Electrochemical impedance spectroscopy (EIS) of the cement/sulfur cathode composite (**a**) before and (**b**) after cycling for 200 cycles at a C/10 rate. Cyclic voltammetry (CV) measurements of the (**c**) reference cathode and (**d**) cement/sulfur cathode composite and the corresponding lithium-ion diffusion coefficient of the (**e**) reference cathode and (**f**) cement/sulfur cathode composite.

Figure 3 summarizes the electrochemical characteristics of the lithium–sulfur cell with the cement/sulfur cathode composite. In Figure 3a, EIS analysis showed the resistances of 437 and 233 ohms in the cell with the cement/sulfur composite and reference cathodes, respectively. Both the cement/sulfur composite and the reference cathodes displayed a similar high slope at the low-frequency region, indicating a smooth lithium-ion diffusion capability. These findings suggest that employing a cement/sulfur cathode composite with insulating cement in the cell results in an elevated resistance when compared to a cathode rich in conductive carbon. However, this increase in resistance was not substantial and did not hinder lithium-ion diffusion [37,38]. To prove this, the cycled EIS data of both the cement/sulfur composite and reference cathodes were analyzed. A cycled lithium–sulfur cell would show the reaction of lithium ions with sulfur, which results in a decrease in both the cell resistance and the lithium-ion transfer. This is because of the polysulfide generation and the sulfide redeposition [39,40]. In Figure 3b, the cycled cement/sulfur composite and reference cathodes showed electrochemical resistances (i.e., charge transfer and interface) of 11.67 and 1.72 ohms and 3.99 and 1.53 ohms, respectively. Both the reference and cement/sulfur cathode composites showed low cell resistance. This is because a cycled lithium–sulfur cell would show the reaction of lithium ions with sulfur and the resulting polysulfide generation and sulfide redeposition, which result in a decrease in both the cell resistance and the lithium-ion transfer, respectively. The relatively low electrochemical resistances obtained from the cycled cement/sulfur composite further indicate that the use of cement for polysulfide adsorption did not affect cell impedance during cycling [37,38,39]. The decrease in overall impedance of the cement/sulfur cathode composites further indicated the diffusing polysulfides that were stabilized within the cathode region and in contact with the added cement and current collector [37,38,39,40], facilitated by the cement’s strong polysulfide-trapping capability. 

CV measurements demonstrated the potentiodynamic redox reactions of the lithium–sulfur cell with the cement/sulfur cathode composite and reference sample. The reference cell showed the typical lithium–sulfur redox reaction with two cathodic reactions and two overlapping anodic reactions. The cathodic reactions resulted in two peaks at 2.22–2.23 V and 1.92–2.03 V, corresponding to the reduction reaction from sulfur to long-chain Li_2_S_4_ (i.e., cathodic-1) and Li_2_S_2_/Li_2_S (i.e., cathodic-2) conversion, respectively. The anodic reaction featured an overlapping peak at 2.47–2.49 V (i.e., anodic), indicating the reversible reaction from sulfide to polysulfide and sulfur. This phenomenon arose from the different conversion speeds during lithium–sulfur cell cycling. During the discharging process, the facile dissolution of sulfur and the formation of polysulfides occurred at the cathodic-1 peak, while the sluggish nucleation of sulfide took place at the cathodic-2 peak. The existence of two distinct discharge peaks could be attributed to the relative ease of sulfur-to-polysulfide conversion compared to the more sluggish polysulfide-to-sulfide conversion. During the charging process, the initial step involved the sluggish dissolution of sulfide, followed by the nucleation of sulfur as the second step. The introduction of LiNO_3_ in the commonly-used lithium–sulfur battery electrolyte served to improve sulfur nucleation [4,39]. This enhancement resulted in an obvious large polarization during the first step, leading to an overlap with the second step featuring a lowered polarization. Thus, this overlap manifested as an anodic peak characterized by two closely aligned peaks in the electrochemical profiles [37,38,39]. As the scanning rate increased, the reference cell showed an increased current density and polarization because of the high rate and resistance. At each rate, the reference cell demonstrated an increase in polarization, suggesting an unstable electrochemical reaction caused by the loss of polysulfides and the formation of an insulating sulfide layer on the cathode (Figure 3c). Conversely, the cell with the cement/sulfur cathode composite displayed redox peaks at 2.20–2.21 V, 1.87–1.99 V, and 2.52–2.53 V. Despite a slight increase in redox polarization observed in the CV peaks, attributed to the substitution of conductive carbon with cement, the characteristic lithium–sulfur redox reactions remained evident. This suggests that the integration of cement into the sulfur cathode as a composite did not significantly influence the cell reaction, causing substantial polarization or irreversible impedance. On the contrary, it contributed to enhancing the electrochemical reversibility and overall stability of the cell [37,38]. As solid evidence, in Figure 3d, as the scanning rate and number increased, the cement/sulfur energy storage material maintained similar low polarization and overlapping CV curves without notable shifts in redox peaks [4,38]. The peak current and corresponding scanning rates were collected from CV analysis and calculated using the Randles–Ševčík equation: i_peak_ = 268,600 × e^1.5^ × area × coefficient_Li-ion_^0.5^ × concentration_Li-ion_ × rate^0.5^. In this equation, i_peak_, e, area, coefficient_Li-ion_, concentration_Li-ion_, and rate represent the peak current, number of electrons, area of the electrodes, lithium-ion diffusion coefficient, lithium-ion concentration in the electrolyte, and scanning rate, respectively. The results revealed coefficient_Li-ion_ values of 2.6 × 10^−8^, 3.6 × 10^−8^, and 3.1 × 10^−7^ cm^2^ s^−1^ for the reference cathode and 2.3 × 10^−8^, 6.8 × 10^−8^, and 1.9 × 10^−7^ cm^2^ s^−1^ for the cement/sulfur cathode composite. This finding further confirmed that the use of cement in the cathode improved polysulfide retention without negatively affecting lithium-ion transfer in the cell (Figure 3e,f) [3,4,37]. These electrochemical analyses and comparisons indicated that the cement/sulfur energy storage material offers enhanced polysulfide retention and reversibility without causing excessive cathode resistance in transfer electrons and lithium ions.

### 3.3. Lithium–Sulfur Cell Performance

**Figure 4:**
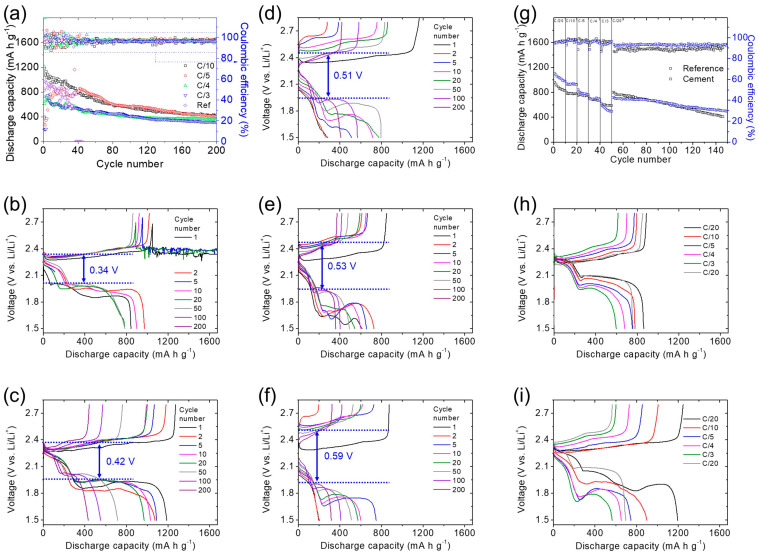
Lithium–sulfur cell performance. (**a**) Cyclability of the cement/sulfur cathode composite at C/10 to C/3 rates and the corresponding discharge/charge voltage profiles of the (**b**) reference cathode at a C/10 rate and the cement/sulfur cathode composite at (**c**) C/10, (**d**) C/5, (**e**) C/4, and (**f**) C/3 rates. (**g**) Rate performance of the cement/sulfur cathode composite at C/20 to C/3 rates and the corresponding discharge/charge voltage profiles of the (**h**) reference cathode and (**i**) cement/sulfur cathode composite.

Figure 4 shows the performance of the lithium–sulfur battery cathode. The cyclability tests showed that the cell with a high sulfur loading of 6.4 mg cm^−2^ and an 80 wt% sulfur content in the cement/sulfur energy storage material achieved high charge storage capacities of 1189, 866, 727, and 716 mA∙h g^−1^ and reversible capacities of 436, 416, 360, and 317 mA∙h g^−1^ at C/10, C/5, C/4, and C/3 rates, respectively, after 200 cycles. This indicates a high electrochemical utilization of sulfur, reaching 71%. Moreover, the cement/sulfur cathode composite in the high-sulfur-loading cathode maintained capacity retention values of 37%, 48%, 50%, and 44% at C/10, C/5, C/4, and C/3 rates, respectively. There is a discernible trend in the calculated capacity retention: as the cycling rate increases from C/10 to C/4 rates, there is an increase in capacity retention, followed by a subsequent decrease at a C/3 rate. This behavior could be attributed to the cement/sulfur cathode composite’s efficacy in serving as a high-loading cathode with high active-material retention over extended cycling periods of 200 cycles. At lower cycling rates, the high-loading cathode experienced a more substantial involvement of active material in the electrochemical reactions, leading to a heightened initial capacity and the generation of a significant amount of polysulfides. This resulted in a comparatively lower capacity retention. Conversely, at higher rates, the conversion process became incomplete due to the large current density, resulting in lower capacity values and a constrained production of polysulfides. While this manifested as a higher capacity retention at high rates, it is noteworthy that the capacity values are typically lower than those observed at lower cycling rates [4,33,34,35]. In addition, considering the use of a high-loading sulfur cathode with a high sulfur content, the resulting cell exhibited high areal and gravimetric capacities of 7.61, 5.54, 4.66, and 4.59 mA∙h cm^−2^ and 951, 693, 582, and 573 mA∙h g^−1^, respectively. Furthermore, a high energy density of 9.63–15.98 mA∙h cm^−2^ was realized. These performance values outperform the requirements for powering electric vehicles and the conventional oxide cathodes in the current commercial lithium-ion batteries [6,31,32,33,34,35,41,42,43,44]. However, the reference cell, which consisted of a large amount of conductive carbon to host polysulfides and enhance cathode conductivity, exhibited rapid and irreversible capacity decay within only 50 cycles (Figure 4a). 

The detailed discharge and charge reactions are summarized in Figure 4b–f. In Figure 4b, the reference cell at a C/10 rate showed two discharge plateaus and one continuous charge plateau with a polarization of 0.34V, which agrees with the findings of CV analysis and corresponds to the two-step discharge reaction from sulfur to polysulfides and sulfides and the continuous charge reaction from sulfide to polysulfide and sulfur, respectively [3,4,37]. However, the reference cathode encountered challenges inherent to lithium–sulfur battery chemistry, including the rapid loss of the active material through fast polysulfide diffusion and increased polarization caused by the unwanted redeposition of diffusing polysulfides on the cathode surface, forming an inactive insulating layer [9,10,11]. In Figure 4c–f, with the assistance of insulating cement that served as the host material in the cathode to trap diffusing polysulfides in the high-loading cathode, at C/10 to C/3 rates, the lean-electrolyte cell with the cement/sulfur cathode composite exhibited slightly high polarization. The polarization values were 0.42, 0.51, 0.53, and 0.59V at C/10, C/5, C/4, and C/3 rates, respectively. These cells exhibited complete discharge and charge plateaus, along with overlapping discharge/charge curves, over the course of 200 cycles. The complete discharge and charge plateaus indicated the enhanced electrochemical utilization of the active material. The incorporation of cement in the cement/sulfur cathode composite contributes to stabilizing the active material within the cathode, enabling the high-loading cathode to sustain high discharge capacities over an extended cycle life of 200 cycles at C/10–C/3 rates [4,5,6]. Additionally, the complete and overlapping charge curves and discharge curves suggested improved electrochemical efficiency, attributed to the inhibition of the polysulfide diffusion and the derived overcharging issues. Moreover, the stabilized polysulfides exhibit strong reaction activity, enhancing the redox conversion of the cell across various rates [9,17,18,19]. In addition, the 200-cycle lifespan of the lean-electrolyte lithium–sulfur cell with a high-sulfur-loading cement/sulfur cathode underscores the electrochemical reversibility achieved in our work [4,9,26]. Therefore, these positive features confirmed the improved electrochemical utilization, efficiency, and reversibility [17,18,19,26].

In addition to the cyclability test, the rate performance of the cells was examined at C/20, C/10, C/5, C/4, and C/3 rates to study the reaction kinetics. Subsequently, the cells were set back to C/20 for assessing electrochemical reversibility. At various cycling rates, the reference cells experienced continuous capacity loss and eventually failed with low discharge/charge efficiency (Figure 4g,h). Conversely, the cement/sulfur cathode composites demonstrated improved rate performance, yielding discharge capacities of 1105, 942, 848, 711, and 614 mA∙h g^−1^, respectively. The sustained high sulfur utilization confirmed the cement’s effectiveness in retaining polysulfides in the cathode as an active material, facilitating excellent reaction kinetics. In rate performance analysis, the cement/sulfur cathode composite showed a reversible capacity of 723 mA∙h g^−1^ and a long cycle life of 100 cycles at a C/20 rate. This finding indicates the improved rate performance and electrochemical reversibility (Figure 4g,i).

**Figure 5:**
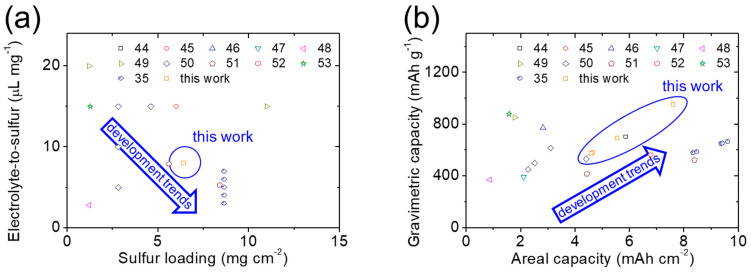
Lithium–sulfur cell performance. (**a**) Cell fabrication parameters: sulfur loadings and electrolyte-to-sulfur ratios and (**b**) cell performance values: areal capacities and gravimetric capacities of cathodes.

Figure 5 and Table 1 investigate the critical cell fabrication parameters, encompassing the loading and content of the active material in high-loading sulfur cathodes, as well as the electrolyte-to-sulfur ratio in lean-electrolyte cells. The reported findings included the resulting values of cell performance and the specific oxide additives that were utilized. The cement/sulfur energy storage material exhibited an impressive capacity to accommodate a high sulfur content, reaching up to 80 wt%, particularly in high-loading sulfur cathodes with 6.4 mg cm^−2^ sulfur. The integration of the high-loading cathode in lean-electrolyte cells further showcased exceptional areal capacity, gravimetric capacity, and an extended cycle life of 200 cycles at various cycling rates [4,35,44,45,46,47,48,49,50,51,52,53]. This suggests that the cement/sulfur energy storage material adopting the conventional composite cathode preparation effectively amalgamated two widely available and cost-effective materials within a sulfur cathode, achieving notable advancements in both cell fabrication parameters and cell performance values simultaneously [14,15,16,17,18,19,35,44,45,46,47,48,49,50,51,52,53]. 

## 4. Conclusions

A cement/sulfur energy storage material with robust polysulfide retention properties was developed in the study. Low-cost cement was used as the host substrate for cost-effective sulfur to prepare the cement/sulfur cathode composite with a high sulfur content and loading of 80 wt% and 6.4 mg cm^−2^, respectively. This cathode design adopted the strong polysulfide retention contributed by cement and resulted in a high charge storage capacity and rate performance, while minimizing the influence of the resistance attributed to cement. Therefore, the cell with the cement/sulfur energy storage material demonstrated a long cycle life exceeding 200 cycles, a high rate performance from C/20 to C/3 rates, and exceptional specific capacity performance. The findings of this study shed light on the polysulfide retention and utilization mechanisms of cement, paving the way for novel material and cathode configuration designs and electrolyte development.

## Figures and Tables

**Table 1:** Cell fabrication parameters and cell performance values of lithium–sulfur cells optimized with functional oxides as additives.

Sulfur Loading (mg cm^−2^) and Sulfur Content (wt%)	Electrolyte-to-Sulfur Ratio (µL mg^−1^)	Peak Capacity (mA·h g^−1^) and Cycling Rate	Areal Capacity (mA·h cm^−2^)	Gravimetric Capacity (mA·h g^−1^)	Cycle Life	Additive	Ref.
8.6/60	7	1280 @ 0.1C	11.1	768	100	cement	35
8.6/60	6	981 @ 0.1C	8.5	589	100	cement	35
8.6/60	5	1127 @ 0.1C	9.7	676	100	cement	35
8.6/60	4	1097 @ 0.1C	9.5	658	100	cement	35
8.6/60	3	1115 @ 0.1C	9.6	669	100	cement	35
5.0/60	--	1172 @ 0.1C	5.9	703	100	TiO	44
6.0/50	15	1130 @ 0.1C	6.8	565	100	Mn_3_O_4_	45
2.0/55	--	1409 @ 0.2C	2.8	773	50	TiO_2_	46
3.0/56	--	703 @ 0.1C	2.1	394	200	TiO_2_	47
1.2/52	2.8	714 @ 1.0C	0.9	371	100	TiO_2_	48
1.2/57	20	1481 @ 0.5C	1.8	851	100	MXene@TiO_2_	49
4.6/56	15	957 @ 0.1C	4.4	533	100	(Mg_0.2_Mn_0.2_Ni_0.2_Co_0.2_Zn_0.2_)Fe_2_O_4_	50
5.6/53	7.9	491 @ 0.1C	4.3	417	1	La_0.8_Sr_0.2_(Cr_0.2_Mn_0.2_Fe_0.2_Co_0.2_Ni_0.2_)O_3_	51
8.4/53	5.3	998 @ 0.1C	8.4	526	1	La_0.8_Sr_0.2_(Cr_0.2_Mn_0.2_Fe_0.2_Co_0.2_Ni_0.2_)O_3_	51
1.7/--	--	1146 @ 0.1C	1.9	--	300	Co_0.08_Mn_0.08_Ni_0.08_Fe_1.96_Mg_0.08_Nd_0.01_Gd_0.01_Sm_0.01_Pr_0.01_O_4_	52
1.3/70	15	1256 @ 0.1C	1.6	879	100	(Ni_0.2_Co_0.2_Mn_0.2_Cu_0.2_Zn_0.2_)WO_4_	53
6.4/80	8	1189 @ 0.1C	7.6	951	200	cement	This work
6.4/80	8	866 @ 0.2C	5.5	692	200	cement	This work
6.4/80	8	727 @ 0.25C	4.7	582	200	cement	This work
6.4/80	8	716 @ 0.33C	4.6	573	200	cement	This work

## Data Availability

Dataset available on request from the authors.
